# The American Institute of Homœopathy; Report of Its Proceedings

**Published:** 1892-08

**Authors:** 


### THE AMERICAN INSTITUTE OF HOMŒOPATHY.

### Report of its Proceedings.

(Condensed from *The Washington Post.*)

The forty-fifth Annual Session of the American Institute of Homœopathy was held June 13th, 14th, 15th, and 16th, 1892.

The hall in which the meetings were held had been handsomely decorated for the occasion with the national colors by two seamen from a U. S. cruiser.

President Theodore Y. Kinne was in the chair.

Dr. McClelland presented the report of the Programme Committee, and Dr. Pemberton Dudley, the General Secretary, reported the work of the Executive Committee. The Treasurer, Dr. E. M. Kellogg, reported an expenditure of $5,610 last year, and a balance on hand of $817.22. Dr. T. Franklin Smith reported on Organization, Registrations, and Statistics. Verbal reports of delegates from different States were also received.

Dr. Tulio S. Verdi offered a resolution for the encouragement of a high standard of medical education in the United States, providing "that the President, by and with the advice and consent of the Senate, shall appoint a board of medical examiners composed of eight members learned in the science and art of medicine and surgery, whose duty it shall be to examine candidates for the degree of United States Master of Medical Science (U. S. M. M. S.)." The bill further provides that the President shall through diplomatic channels secure the recognition of this degree in foreign countries, where the American medical diploma is now looked upon with disfavor if not absolute scorn.

Addresses were presented of the bureaus of Ophthalmology, Otology, and Laryngology, by Dr. A. B. Norton, of New York; Obstetrics, by Dr. George B. Peck, of Providence; and Gynæcology, by Dr. M. T. Runnels, of Kansas City. Dr. J. D. Buck reported a list of twenty-three homœopathic publications. Dr. E. F. Storke, of Denver, reported on Foreign Correspondence.

In the evening of the first day's session a brilliant reception was held at the New National Theatre. At this reception a masterly address was delivered by Dr. Kinne, which we have not space to print.

At the session next day a committee was appointed upon the President's address, and then an amendment was made to the Constitution by the election of two instead of one Vice-President.

Dr. B. W. James, acting chairman of the bureau of Sanitary Science, reported the annual address of Dr. Beckwith, of Cleveland. This address stated in brief that one of the greatest needs of our highly developed modern civilization is a perfect and general sanitation.

Dr. McClelland offered a motion for the appointment of a committee upon the erection of a monument to Samuel Hahnemann. It was unanimously carried, and a committee was appointed consisting of Drs. J. H. McClelland, J. B. Dake, I. T. Talbot, J. S. Mitchell, and H. M. Smith.

On motion of Dr. Henry M. Smith, of New York, a resolution was passed providing for a button as a badge of the Institute.

A resolution was also passed providing for a special committee to be devoted to forwarding the interests of Homœopathy at the World's Medical Congress, at Chicago, in connection with the World's Columbian Exposition, next year.

There was a paper upon Javal Ophthalmometer, by Dr. C. M. Thomas; Exophthalmic Goitre, by Dr. E. H. Linnell; Antisepsis and Asepsis in Ophthalmic Surgery, by Dr. Harold Wilson, of Detroit; Paralysis of all the ocular muscles except the sphincter iridis, by Charles H. Helfrich, M. D., of New York; Heterophoria with Cases, by Hayes C. French, M. D., San Francisco; The Eye in General Diagnosis, by Thomas M. Stewart, M. D., Cincinnati; Herpes Zoster Ophthalmicus, by D. A. MacLachlan, M. D., Ann Arbor; Aural Therapeutics, by Henry C. Houghton, M. D., New York. But the most remarkable paper was entitled "Massage of the sound-conducting apparatus of the Ear by means of vibratory force and the similar sound as a curative agent in tinnitis aurium," by Henry F. Garey, M. D., of Baltimore. In this machine vibratory force or motion is made to act upon the sound-conducting parts of the ear, producing massage, thereby relieving those conditions which before could not be reached by the regular modes of treatment and which were the regular causes of deafness in a very large proportion of those afflicted. The phonograph was at first used to bring about these results, but lately an instrument which has been named the vibrometer has been devised for this special purpose which makes this mode of treatment more effective. The doctor then reported the results in a number of persons treated. Some whose deafness was from five to fifteen years'

standing could now hear ordinary conversation from ten to twenty feet away with their back turned to the speaker, and others with never-ceasing noises in their ears were completely relieved.

There was a sectional meeting of the Bureau of Obstetrics at which the Chairman, Dr. George B. Peck, presided. The following papers were presented: "Post-partum Treatment," by C. H. Cogswell, M. D., Cedar Rapids, Iowa; "Cleanliness in Obstetrics," by C. A. Pauly, M. D., Cincinnati; "Asepsis *versus* the Colored Nurse," by Sarah J. Millsop, M. D., Bowling Green, Ky.; "Obstetric Antisepsis," by L. L. Danforth, M. D., New York; "The Better Way," by George W. Winterburn, M. D., New York; "The Practical Relations of the Homœopathist to the Germ Theory," by George B. Peck, M. D., Providence, R. I.

Dr. A. W. Woodward, of Chicago, presented a report entitled: "A Series of Experiments with Cinchona, Ipecac, Pulsatilla, and Rhus-tox., made for the purpose of learning the common sequence of effects produced by each of these drugs upon the healthy body."

The Committee on Life Insurance Examiners also reported. The report showed that while a few companies still held out, over two-thirds of all the life insurance organizations of the country now employ homœopathic examiners.

Dr. Millie J. Chapman read the report of the Bureau of Pedology. The report of the Committee upon Medical Education was given by Dr. Fisher, of Texas. The Institute had decreed three years ago that after 1891 no college graduating students on less than three courses of six full months would be recognized by the Institute, nor would such graduates be admitted to membership in it. All the homœopathic institutions had fulfilled the requirements to the letter, and the two courses of lectures is a thing of the past. Papers were presented by Dr. T. G. Comstock, of St. Louis, on "Shall our Colleges adopt the Recitative Plan of Teaching?" by Dr. Orne, of Atlanta, Ga., "The Hospital should precede the College;" Dr. Spaulding, of Boston, on "Private Pupilage;" Dr. Talbot, of Boston, "A Plea for Better Methods in Medical Teaching," etc.

A resolution was adopted "that the American Institute would look with decided disapprobation upon the organization of college faculties in cities where ample hospital and clinical facilities shall not have been previously provided." The report of the Committee on Legislation was presented. It has been doubtful if homœopathists were admissible for examination for admission to the army and navy, owing to the special regulation that no one should be admitted to such examination who was not a graduate of some "regular school." Upon inquiry the surgeon-general stated that the meaning of the rule made by the Department was not exclusive in intention and the term "regular" was used simply as meaning a well-equipped medical college requiring not less than a three years' course to secure its diploma. "This lets in Homœopathy," said Dr. Orme, "as all our colleges are well equipped and require a three years' course before graduation. Therefore our practitioners are by reason of this important decision upon an equal footing with all others in entering the army and navy."

The officers elected were as follows: President, Dr. James H. McClelland, of Pittsburgh, Pa.; First Vice-President, Dr. C. E. Fisher, of San Antonio, Texas; Second Vice-President, Dr. Millie J. Chapman, of Pittsburgh, Pa.; Treasurer, Dr. E. M. Kellogg, of New York city; Assistant Treasurer, Dr. T. F. Smith, of New York; General Secretary, Dr. Pemberton Dudley, of Philadelphia; Provisional Secretary, Dr. T. M. Strong, of Boston; Board of Censors, Drs. Rush, Cowperthwaite, Smith, Hoag, and Kenyon. Dr. H. M. Smith, of New York, was appointed necrologist. Chicago was chosen as the next place of meeting.

A resolution was offered, calling the attention of the Senate of Seniors to the practice of certain members using secret and proprietary medicines, and advertising themselves as so doing, and requesting that they take whatever action they shall deem proper.

The report of the Senate of Seniors is an official proclamation that patent medicine men and specialists who belong, or claim to belong, under the particular protection of Homœopathy will be summarily ousted from its ranks, if the charge of charlatanry is established against them.

The report positively forbids any homœopathic physician from advertising himself possessed of any remedy or method of cure not known, and capable of being used by the entire medical profession, emphasizing that the physician should depend for his standing upon his able judgment and training, and not upon discovering quack cures.

In the Bureau of Materia Medica the following papers were presented: "The Evolution of Materia Medica," by W. E. Leonard, M. D., Minneapolis, Minn.; "The Teaching of Materia Medica in Homœopathic Colleges," by Richard Hughes, M. D., Brighton, Eng., honorary member; "Proving of Saw Palmetto on a Woman," by Will S. Mullins, M. D., Lexington, Ky.; "What are the Laws of Cure?" by M. W. Vandenburg, M. D., Fort Edward, N. Y.; "Proving of Ficus Indica," by Dr. B. N. Banerjee, Calcutta, India; "The History, Synthetic Symptomatology, Therapeutic Application, and Comparison of Thuya Occidentalis," by the Medical Investigation Club, of Baltimore, Md.

The following papers were contributed to the Bureau of Surgery: "General Considerations of Visceral Surgery," by Charles E. Walton, M. D., of Cincinnati, Ohio; "Appendicitis, Diagnosis and Treatment," by A. Boothby, M. D., Boston, Mass.; "The Treatment of Pulmonary Abscess," by W. F. Knoll, M. D., Chicago, Ill.; "Surgery of the Gall-Bladder," by Horace Packard, M. D., Boston, Mass.; "Surgery of the Kidney," by George F. Shears, M. D., Chicago, Ill.; "Laparotomy in the Treatment of Epilepsy," by S. B. Parsons, M. D., St. Louis, Mo., and "Prolonged Abdominal Irrigation in Overcoming Tumor Adhesions," by M. O. Terry, M. D., Utica, N. Y.

The foregoing is but an outline of the work done by the Institute. Its business was enormous, and it would be impossible to find room for all the minutes in these pages.

